# Reduction in *Pseudomonas aeruginosa* sputum density during a cystic fibrosis pulmonary exacerbation does not predict clinical response

**DOI:** 10.1186/s12879-015-0856-5

**Published:** 2015-03-22

**Authors:** John C Lam, Ranjani Somayaji, Michael G Surette, Harvey R Rabin, Michael D Parkins

**Affiliations:** Department of Medicine, The University of Calgary, 3330 Hospital Dr. NW, Calgary, Alberta T2N 4 N1 Canada; McMaster University, Hamilton, Ontario Canada; The Department of Microbiology, Immunology and Infectious Disease, The University of Calgary, Calgary, Canada

**Keywords:** Treatment, Biomarker, Failure, Outcome, Bronchiectasis, Cefepime, Heterogeneity

## Abstract

**Background:**

Pulmonary exacerbations (PEx) are critical events in cystic fibrosis (CF), responsible for reduced quality of life and permanent loss of lung function. Approximately 1/4 of PEx are associated with failure to recover lung function and/or resolve symptoms. Developing tools to optimize PEx treatment is of paramount importance.

**Methods:**

We retrospectively audited all adults infected with Pseudomonas aeruginosa, experiencing PEx necessitating parenteral antibiotic therapy from 2006–2012 from our center. Quantitative analysis of sputum at admission, twice-weekly during hospitalization, and end of therapy were compared to baseline (most recent healthy) and follow-up (after PEx) samples. Change in P. aeruginosa burden from baseline was assessed for any and all morphotypes (ALL), as well as mucoid (MUC) and non-mucoid (NON) isolates specifically. PEx were identified as failures if >90% of baseline pulmonary function was not recovered.

**Results:**

Forty-six patients meeting the above inclusion and exclusion criteria experienced 144 PEx during this time (median 3, IQR 2–6). Patients were treated for a median 14 days (IQR 13–16). No increase in ALL, MUC or NON were detected at PEx, nor was there an association between change in sputum density and magnitude of lung function decline. PEx failures were observed in 30% of events. Reductions of at least 1-log and 2 log P. aeruginosa sputum density was observed in 57% and 46% (ALL), 73% and 55% (MUC) and 58% and 46% (NON) of PEx, respectively. Factors associated with greater reduction of P. aeruginosa sputum density included choice of β-lactam antibiotic, antibiotics with in vitro predicted activity and treatment duration. PEx associated with reductions in P. aeruginosa sputum density were not associated with a reduced risk of PEx failure.

**Conclusions:**

Enhanced killing of P. aeruginosa during PEx does not predict improved clinical outcomes. Studies accounting for the polymicrobial nature of CF respiratory disease and the heterogeneity of P. aeruginosa causing chronic infection may enable the identification of a more appropriate pathogen(s) based biomarker of PEx outcomes.

## Background

The archetypal CF pathogen, *Pseudomonas aeruginosa* infects 50-70% of patients [1]. Patients with chronic *P. aeruginosa* infection have increased rates of lung function decline, health care utilization, and reduced survival [2-4]. Chronic *P. aeruginosa* infection is punctuated by frequent acute deteriorations termed pulmonary exacerbations (PEx). PEx are characterized by increased cough and sputum production, disproportionate shortness of breath and loss of lung function, as well as increased inflammation [5-7]. PEx are critical events in CF, associated with reduced quality of life [8], increased cost [9], permanent lung damage [10,11] and increased short-term mortality [12,13]. So important are these events, they may now constitute primary end-points in CF therapeutic trials [14].

Treatment of PEx usually consists of aggressive airway clearance, respite, nutritional support, and antimicrobial therapy directed against chronically infecting pathogens. Despite therapy, 25% of PEx fail to achieve successful outcomes as determined by lung function recovery, resolution of symptoms and preventing recurrences [15]. Patients more likely to experience unsuccessful PEx outcomes are infected with MRSA, *Burkholderia cepacia complex,* MDR (multi-drug resistant) *P. aeruginosa*, and those with CF-related diabetes and/or CF-liver disease [10,15]. Furthermore, more severe PEx including those with greater initial lung function decline, and those with greater inflammation at initiation and completion of treatment are associated with worse outcomes.

Developing tools to predict PEx outcomes is key to their successful management. It has been previously determined that antibiogram directed therapy against a limited number of isolates of *P. aeruginosa* has only a weak association with PEx outcomes [15]. As such, other biomarkers are increasingly being evaluated for their ability to predict treatment responses. A number of host specific factors are being assessed [16,17]. However, given the critical intervention in PEx is anti-bacterial, the use of bacterial derived biomarkers to follow treatment response deserves attention. While antibacterials have been shown to reduce the bacterial load during the treatment of PEx, how this correlates with clinical response has not been established [18]. Herein we evaluate the use of semi-quantitative reporting of *P. aeruginosa* sputum density and correlated the response with clinical outcomes during PEx treatment.

## Methods

All CF patients chronically infected with *P. aeruginosa* attending the Calgary Adult CF Clinic from 2006–2012 experiencing PEx treated with parenteral antibiotics were considered for inclusion if they had semi-quantitative sputum cultures performed ≥3 times during treatment (baseline, initiation, early, end of therapy) and at follow-up. Parenteral antibiotics provided for reasons other than PEx were excluded. Patients were excluded if they had a baseline FEV_1_ < 30% predicted, were infected with *Burkholderia cenocepacia* or *Mycobacterium abscessus,* or were listed for lung transplantation. Detailed review of clinical records were performed from prior to the PEx, through treatment and in follow-up. Pulmonary function was evaluated by spirometry. Data was prospectively collected and retrospectively analyzed. Patients provide prospective consent for studies correlating clinical outcomes with lower airways infection. This project has been approved by the Conjoint Health Research Ethics Board of the University of Calgary (E23087).

### Definitions

Baseline lung function was defined as the best forced expiratory volume in one second (FEV_1_) percent predicted in the preceding 6 months. The Leeds criteria was used to define chronic *P. aeruginosa* infection [19]. PEx were diagnosed on clinical grounds in real time, however, charts were evaluated and scored according to Fuchs criteria retrospectively [5]. Parenteral therapies were provided as either an inpatient, outpatient via the home parenteral therapy program (HPTP) or a combination thereof. Treatment failures were defined *a priori* as failure to have recovered 90% of baseline FEV_1_ at follow-up (FTR90) [10,11,15]. Severity of lung disease was categorized as mild (FEV_1_% predicted ≥70%), moderate (40-70%), and severe (<40%). Bacterial sputum density is reported as log10 CFU/ml. Bacteriologic responses to anti-pseudomonal therapies were evaluated as either a one or two-log drop in CFU/ml.

### Antibacterial therapies

Anti-Pseudomonal β-lactams (aztreonam, ceftazidime, cefepime, and meropenem) were dosed 2 g every 8 hours infused over thirty minutes, ciprofloxacin as 600 mg IV or 750 mg PO every 12 hours, and tobramycin as 10 mg/kg/day divided in 1–2 doses. When patients with chronic *Staphylococcus aureus* co-infection were treated with regimens lacking Staphylococcal activity such as aztreonam/ceftazidime, supplemental anti-Staphylococcal therapies were used and consisted of trimethoprim/sulfamethoxazole (TMP/SMX) 320/1600 mg PO BID, linezolid 600 mg PO BID, rifampin 300 mg PO BID, or cloxacillin 2 g IV q4h, clindamycin 600 mg IV q8h, ceftriaxone 2 g IV q12h or tigecycline 50 mg q12h.

### Microbiologic investigations

Baseline sputum samples were from the most recent clinic where patients were not experiencing PEx. Twice-weekly sputum samples were collected through treatment. Admission samples were collected before starting therapy. Changes in bacterial load were evaluated at “EARLY” (3^+/−^2 days from starting) and “END” (within 3 days of completion). Follow-up samples were collected at the next clinic visit attended.

Sputum samples were mixed with equal volumes of dithiothreitol (DTT, Sputolysin, EMD Millipore, MA) and vortexed. Samples were serial diluted in DTT to 10^−5^ and plated on Columbia Blood Agar (CBA), MacConkey Agar, (MAC), Chocolate Agar (CHOC), *Burkholderia cepacia* selective agar, and Mannitol Salt Agar. Pathogens were identified by standard criteria [20]. Oral flora (OF) included organisms such as non-hemolytic Streptococci, *Haemophilus parainfluenzae*, *Corynebacterium* spp., *Neisseria* spp. (non-*meningitidis*) and Coagulase-negative Staphylococci grown on CBA or CHOC. Organisms were quantified and *P. aeruginosa* colonies classified as mucoid (MUC) or non-mucoid (NON) isolates based on growth on MAC [21]. *P. aeruginosa* susceptibility testing was performed on each morphotype by Kirby-Bauer or E-test as per CLSI guidelines [22]. Where >1 MUC/NON isolates existed susceptibility testing was performed on each, and the result represents the least susceptible. MDR was defined as resistance to all antibiotics in ≥2 classes and pan-drug resistance (PDR) all classes, save for colistin [15,23].

### Statistical analysis

Means with standard deviation (SD) or medians with inter-quartile ranges (IQR) were used to describe normal and non-normally distributed data, respectively. Categorical variables were compared using a two-sided Fisher’s exact test. Odds ratios (OR) were calculated by dividing the proportion with a factor to those without and were reported with 95% confidence intervals (CI). Continuous variables were compared using Pearsons correlation coefficient. Statistical analyses were performed using Stata 12.0 (Stata Corp., College Station, TX, USA). A p value of ≤0.05 was considered significant.

## Results

### Patients and PEx

Of a total eligible cohort of 176, 46 patients met inclusion criteria. These 46 patients experienced 144 PEx during the six-year study period. Median age of those included was 32.9 years (IQR 27.9-40). Patients were diagnosed with CF at a median age of 1.35 years (IQR 0.5-6). 56% were F508del homozygous and 83% carried ≥1 allele. 43 patients (93%) were pancreatic insufficient. Co-morbidities included CF-related diabetes in 18 (40%), CF-liver disease 8 (18%), osteopenia 60% and osteoporosis 9%. During the six years of study, 14 patients initially included had future events excluded because of progression of disease and three patients died, although none from CF.

Patients experienced a median 3 exacerbations (IQR 2–6). Time to next PEx was 207 days (IQR 93–458). Complete details were not available for all. Using Fuchs criteria to confirm the clinical diagnosis of PEx, patients had a median of 5 criteria (IQR 5–7), where 4 meet the definition. The most common criteria were increased cough (99%) and sputum (99%), increased dyspnea (98%), decreased FEV_1_ by ≥10% (94%), weight loss or anorexia (77%), change in chest exam (72%), radiographic infiltrates (52%), lethargy (46%), fevers (37%), and hemoptysis (36%). Sinus/nasal discharge symptoms were uncommonly documented (2%). At the time of exacerbation, chronic medications included: azithromycin 56%, H2-blockers or proton-pump inhibitors 37%, inhaled corticosteroids (ICS) 54%, long acting bronchodilators 85%, nebulized DNase 66%, tobramycin inhalation solution 54%, and aztreonam solution for inhalation 4%. With the exception of inhaled antibiotics, chronic medications were continued during PEx. 70 (48%) of events were exclusively managed in hospital, 42 (29%) were treated with HPTP, 32 (22%) started off in hospital but completed at home, and two (1%) were admitted to hospital from HPTP. Exacerbations were associated with a median 19.3% (IRQ 11–28.5) and 18.2% (9.9-29) drop in FEV_1_ and FVC respectively.

Patients were treated for a median 14 days (IQR 13–16). Two anti-pseudomonal antibiotics were used in 98% of exacerbations, and three in 2%. These generally consisted of an anti-pseudomonal β-lactam and tobramycin (96%). Ceftazidime was used in 51%, meropenem 24%, cefepime 16%, and aztreonam 7%. Rarely ciprofloxacin was combined with tobramycin (2%). Tobramycin was used in once (14%) or twice-daily doses (81%), and parenteral ciprofloxacin was used in place of tobramycin in five (3%) exacerbations. Additional anti-Staphylococcal therapy was used in 37 (26%) PEx and consisted of TMP/SMX (38%), cloxacillin and TMP/SMX (19%), cloxacillin and rifampin (14%), cloxacillin (11%) and rifampin, clindamycin, clindamycin and ceftriaxone, tigecycline and linezolid each in 3%.

Patients presented with an exacerbation 68 days (IQR 32–111) after their most recent clinic visit. Mean duration from initiation of antibiotics to collection of EARLY sputum culture was 3.84 days (SD 1.75). Mean duration from the end of therapy to collection of END sputum sample was 0.72 days (SD 1.70). 40 days (IQR 27–61) after completion of PEx treatment, patients were seen in follow-up at a regular CF clinic.

### PEx outcomes

PEx treatment failures, as defined by failing to recover 90% of baseline FEV_1_, were observed in 42/140 (30%). Neither sign, symptom, nor total Fuchs’ initial PEx diagnostic score were associated with increased failure risk (not shown). Stage of CF-lung disease did not influence failure (not shown). Antibiotic courses ≤10 days were more likely to be deemed failures; 8/16 (50%) vs 38/128 (30%), OR 1.68 (0.97-2.94), p = 0.15) but this did not reach statistical significance. Few prior and concomitant medications influenced PEx outcomes. In particular, prior tobramycin inhalation solution was associated with a trend to reduced risk of failure [17/74 vs 24/63 OR 0.6 (CI 0.36-1.01), p = 0.06] whereas concomitant ICS were associated with a trend to increased risk [27/42 vs 47/97, OR 1.33 (CI 0.98-1.80), p = 0.09] of failure. Antibiotic choice did not impact PEx outcome, nor did the number of antibiotics with *in vitro* predicted activity against ALL, MUC or NON (not shown). Failure risk did not differ based on delivery of PEx treatment; 33/68 (49%) in hospital, 18/42 (43%) HPTP, 9/32 (28%) transitioned from hospital to HPTP, 1/2 (50%) admitted to hospital from HPTP, p = 0.24. Concurrent use of additional anti-staphylococcal agents did not reduce failure risk [11/30 (36%) vs 31/110 (28%), OR 1.32 (CI 0.75-2.31), p = 0.37].

### Bacteriologic response to antibiotics during PEx

**Figure 1:**
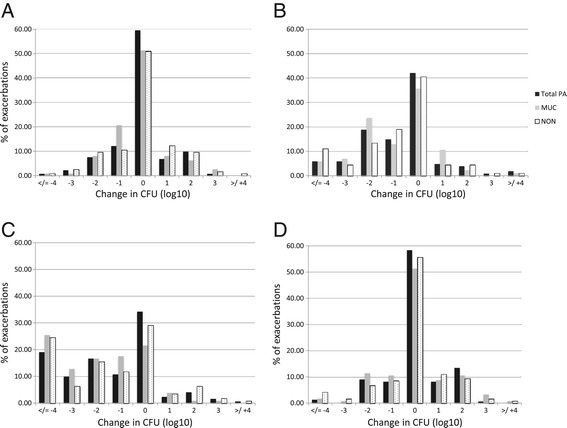
**Change in** ***P. aeruginosa*** **CFU/ml during PEx. A)**. Change in CFU from baseline sputum to admission at time of PEx. **B)**. Change in CFU at EARLY time point (~day 4) of antibiotic treatment relative to admission. **C)**. Change in CFU at the END of antibiotic treatment for PEx relative to admission. **D)**. Change in CFU at follow-up clinic visit relative to admission. Black bars represent all *P. aeruginosa* isolates, grey bars only MUC, and white bars with black dots NON.

Median sputum density of OF at PEx was 10^6^ CFU/ml (IQR 10^5^-10^6^), *S. aureus* 10^6^ (IQR 10^5^-10^6^), *P. aeruginosa* ALL 10^6^ (IQR 10^6^-10^7^), MUC 10^6^ (IQR 10^5^-10^6^) and NON 10^6^ (IQR 10^5^-10^7^). Relative to baseline, admission PEx samples did not demonstrate an increase in *P. aeruginosa* burden (Figure 1A). Furthermore, there was no correlation between the extent of pulmonary function decline at PEx presentation and change in bacterial load (not shown). Treatment produced a progressive decline in *P. aeruginosa* in 60% of patients peaking at end of therapy, but returned to baseline at follow-up (Figure 1B-D). Stage of lung disease during PEx did not influence bacteriologic response (not shown).

Clinical outcomes were not predicted based on changes in sputum bacterial burden at completion of PEx treatment (Table 1). Furthermore, outcomes were not different in those few individuals who transiently cleared ALL, MUC or NON (not shown).

**Table 1:** **Bacteriologic response does not predict clinical outcomes of pulmonary exacerbations**

**Organism**	**Morph**	**Log drop**	Failure risk*		
			Actual values**	**OR (CI)**	**P value**
*P. aeruginosa*	ALL	1	26% (18/68) vs 33% (17/51)	0.78 (0.46-1.38)	0.42
		2	27% (15/55) vs 31% (20/64)	0.87 (0.5-1.5)	0.68
	MUC	1	28% (21/74) vs 23% (6/26)	1.22 (0.56-2.70)	0.79
		2	30% (17/56) vs 22% (10/44)	1.36 (0.68-2.62)	0.49
	NON	1	30% (19/64) vs 35% (16/46)	0.85 (0.49-1.47)	0.68
		2	33% (17/51) vs 30% (18/59)	1.1 (0.63-1.88)	0.84
*S. aureus*		2	46% (12/26) vs 0% (0/3)	n/a	0.25
		4	31% (5/16) vs 54% (7/13)	0.58 (0.28-1.58)	0.27
Oral Flora		1	22% (13/60) vs 34% (23/67)	0.63 (0.35-1.13)	0.12
		2	23% (9/39) vs 31% (27/88)	0.75 (0.39-1.44)	0.52

*Failure was defined as failing to achieve 90% of baseline percent predicted FEV_1_ at time of follow-up.

**Risk of failure to achieve outcome in those PEx achieving the log drop in bacterial counts relative to those events where a drop was not observed.

ALL = all *P. aeruginosa* morphotypes, MUC = mucoid, NON = non-mucoid isolates, OR = odds ration, CI = confidence intervals.

**Figure 2:**
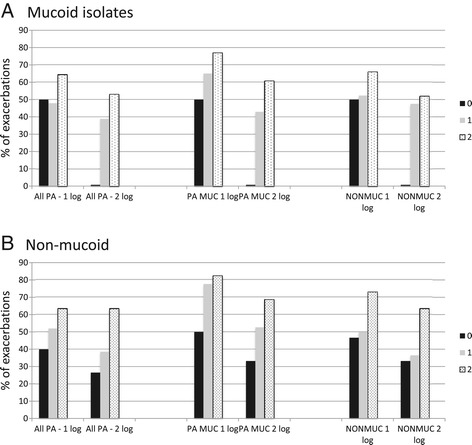
**Percentage of PEx achieving a 1 or 2-log reduction in** ***P. aeruginosa*** **sputum density at end of antibacterial therapy as a function of the number of empirically provided antibiotics with** ***in vitro*** **predicted activity from admission sputum sample. A)**. Antibiotics with activity against Mucoid isolates. **B)**. Antibiotics with activity against Non-Mucoid isolates.

**Figure 3:**
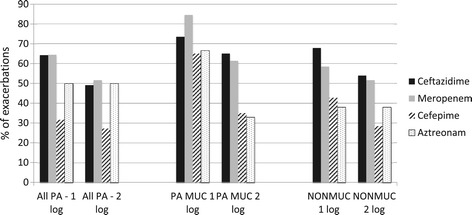
**Percentage of PEx achieving a 1 or 2-log reduction in** ***P. aeruginosa*** **sputum density at end of antibacterial therapy as a function of the β-lactam antibiotic used.**

In PEx where anti-pseudomonal β-lactams were used, 22.5% (32/142) of PEx had ≥1 isolate that was resistant. At least one isolate was resistant to tobramycin, in 54.3% (77/142) of PEx. Highly resistant isolates were rare in admission PEx samples and over a one-year period prior to PEx (MDR; MUC 6% (6/103), 22% (25/116); NON 41% (45/110), 50% (57/113) and PDR; MUC 1% (1/103), 3% (3/115) and NON 12% (13/109), 22% (25/113), respectively. Antibiotic choice impacted *P. aeruginosa* sputum reduction to varying degrees (Table 2, Figures 2 and 3). Relative to other antibiotics ceftazidime showed a modest advantage, whereas cefepime was associated with reduced *P. aeruginosa* killing. Tobramycin dosing once or twice daily did not influence bacteriologic response (data not shown). An association between number of antibiotics with *in vitro* predicted activity and reduction of *P. aeruginosa* density was observed although this reached statistical significance only for regimens with activity against the NON-isolates (Table 3).

**Table 2:** **Treatment factors associated with reduced** ***P. aeruginosa*** **bacterial burden at END of PEx therapies**

**Factor**	**ALL**				**MUC**				**NON**			
	**Value**	**OR (95% CI)**	**Value**	**OR (95% CI)**	**Value**	**OR (95% CI)**	**Value**	**OR (95% CI)**	**Value**	**OR (95% CI)**	**Value**	**OR (95% CI)**
	**1-log reduction**		**2-log reduction**		**1-log reduction**		**2-log reduction**		**1-log reduction**		**2-log reduction**	
Antibiotics*												
FEP vs other ABx	7/22 vs 61/97	0.51 (0.27-0.95), p = 0.009	6/22 vs 49/97	0.54 (0.26-1.08), p = 0.06	13/20 vs 61/81	0.86 (0.61-1.22), p = 0.40	7/20 vs 49/81	0.57 (0.31-1.00), p = 0.05	9/21 vs 55/89	0.69 (0.41-1.16), p = 0.14	6/21 vs 45/89	0.56 (0.28-1.14), p = 0.08
CAZ vs other ABx	36/56 vs 32/63	1.27 (0.93-1.73), p = 0.19	29/56 vs 26/63	1.25 (0.85-1.85), p = 0.27	36/49 vs 38/52	1.00 (0.8-1.27), p = 1.0	32/49 vs 24/52	1.41 (0.99-2.02), p = 0.08	34/50 vs 30/60,	1.36 (0.99-1.86), p = 0.07	27/50 vs 24/60	1.35 (0.90-2.01, p = 0.18
Treatment parameters*												
<14 vs ≥14 days	32/68 vs 29/52	0.84 (0.6-1.2), p = 0.36	23/55 vs 38/65	0.7 (0.5-1.24), p = 0.09	38/74 vs 13/28	1.1 (0.7-1.74), p = 0.8	25/56 vs 26/46	0.78 (0.53-1.16),p = 0.3	30/64 vs 28/46	0.77 (0.54-1.1), p = 0.17	20/51 vs 38/59	0.6 (0.41-0.9), p = 0.01

*Other antibiotics including aztreonam, meropenem, ciprofloxacin and dosing of tobramycin did not show significance and have not been included.

CAZ = Ceftazidime, FEP = Cefepime, ABx = antibiotics.

**Table 3:** **Relation of** ***in vitro*** **predicted susceptibility to reduction in** ***P. aeruginosa*** **bacterial burden at END of PEx therapies**

**Factor**	**ALL**				**MUC**				**NON**			
	**Value**	**OR (95% CI)**	**Value**	**OR (95% CI)**	**Value**	**OR (95% CI)**	**Value**	**OR (95% CI)**	**Value**	**OR (95% CI)**	**Value**	**OR (95% CI)**
	**1-log reduction**		**2-log reduction**		**1-log reduction**		**2-log reduction**		**1-log reduction**		**2-log reduction**	
All Isolates S to β-lactam vs not	59/91 vs 8/27	2.18 (CI 1.2-3.99) p = 0.002	48/91 vs 6/27	2.38 (1.14-4.94) p < 0.01	63/79 vs 10/20	1.59 (1.02-2.51), p = 0.01	48/79 vs 7/20,	1.73 (1.0-3.23) p = 0.05	55/81 vs 8/27	2.29 (1.26-4.18), p < 0.001	44/81 vs 6/27	2.44 (1.17-5.09), p = 0.004
All isolates S to TOB vs not	32/53 vs 35/65	1.12 (0.82-1.53), p = 0.56	29/55 vs 25/65	1.37 (0.92-2.03), p = 0.14	34/46 vs 39/53	1.0 (0.79-1.27), p = 1	28/46 vs 27/53	1.19 (0.84-1.69), p = 0.41	28/45 vs 35/63	1.12 (0.82-1.54), p = 0.56	25/45 vs 25/63	1.4 (0.94-2.1), p = 0.12
Regimen with 2 vs ≤1 MUC agent*	47/73 vs 12/25	1.34 (0.87-2.08), p = 0.16	39/73 vs 9/25	1.48 (0.85-2.61), p = 0.16	57/74 vs 16/25	1.2 (0.88-1.66), p = 0.29	45/74 vs 10/25	1.52 (0.91-2.54), p = 0.1	N/A		N/A	
Regimen with 2 vs ≤1 NON agent*	29/41 vs 33/67	1.43 (1.05-1.96), p = 0.04	26/41 vs 24/67	1.77 (CI 1.19-2.63), p = 0.006	N/A		N/A		30/41 vs 33/67	1.49 (1.09-2.02), p = 0.02	26/41 vs 24/67	1.77 (1.19-2.63), p = 0.006
MDR at PEx* vs not	N/A		N/A		2/5 vs 59/77	0.52 (0.18-1.54), p = 0.1	1/5 vs 46/77	0.33 (0.06-1.95) p = 0.15	19/41 vs 33/47	0.66 (0.45-0.96), p = 0.03	14/41 vs 27/47	0.6 (0.36-0.97), p = 0.03
MDR in prior year* vs not	N/A		N/A		13/18 vs 47/63	0.96 (0.96-0.7-1.3), p = 1	11/18 vs 36/63	1.06 (0.7-1.63), p = 0.8	22/51 vs 30/36,	0.52 (0.36-0.73), p < 0.001	17/51 vs 24/36	0.5 (0.32-0.78), p = 0.003
PDR at PEx* vs not	N/A		N/A		0/1 vs 61/81	n/a p = 0.21	0/1 vs 46/79	n/a p = 0.4	5/13 vs 47/75	0.61 (0.3-1.29),p = 0.13	4/13 vs 37/75	0.62 (0.27-1.45), p = 0.24
PDR in prior year* vs not	N/A		N/A		0/1 vs 59/79	n/a p = 0.26	0/1 vs 46/79	n/a p = 0.43	8/22 vs 43/65	0.55 (0.31-0.98), p = 0.02	7/22 vs 34/65	0.61 (0.32-1.17), p = 0.14

*In these events, bacteriologic response of *P. aeruginosa* was evaluated specifically against the morphotype type that susceptibility was being evaluated.

S = sensitive as defined by CLSI criteria. TOB = tobramycin. MUC = mucoid isolate, NON = non-mucoid isolate. PEx = pulmonary exacerbation. OR = Odds ratio, CI = confidence interval. N/A = not applicable.

MDR/PDR at event = Presence of an MDR/PDR isolate at admission sputum sample of the PEx being evaluated.

MDR/PDR in prior year = Presence of an MDR/PDR isolate at any time-point in the one year prior to the PEx event being evaluated.

No significant impact of concurrent therapies was noted on reductions in *P. aeruginosa* sputum density. In particular, concurrent chronic azithromycin, DNase, nor hypertonic saline impacted *P. aeruginosa* density. A trend was observed in individuals receiving concurrent ICS at having a lower likelihood of reducing *P. aeruginosa* sputum burden (not shown). Venue for antibiotic delivery did not impact bacteriologic response (not shown). Treatments <14 days produced an inferior bacteriologic response, although this met statistical significance for only the NON-isolates (Table 2).

MSSA was a chronic colonizer of patients in 31/146 (21.2%) of PEx, and two had a history of recent-prior MRSA transient colonization, though none on admission. MSSA was more likely to demonstrate a significant bacteriologic response with 26/30 (81%), 20/30 (67%), 14/30 (46%) experiencing at least a 2-log, 4-log or 6-log drop in MSSA, respectively at the end of therapy. Bacteriologic responses of MSSA and OF during PEx did not predict clinical success (Table 1). During fourteen PEx, new pathogens were apparent during twice-weekly samples, different from admission. These included Aspergillus (4), *Stenotrophomonas maltophilia* (4), Group C Streptococcus (3), and one each of MSSA, MRSA and *H. influenzae*. None of these resulted in changed antimicrobial management (with the exception of TMP/SMX for MRSA) and none manifested in chronic infection. Exacerbations associated with the emergence of new pathogens did not impact risk of PEx failure [5/42 (12%) vs 9/108 (8%), OR 1.41 (CI 0.5-3.98), p = 0.54].

### Early bacteriologic response and correlation to end of therapy

**Figure 4:**
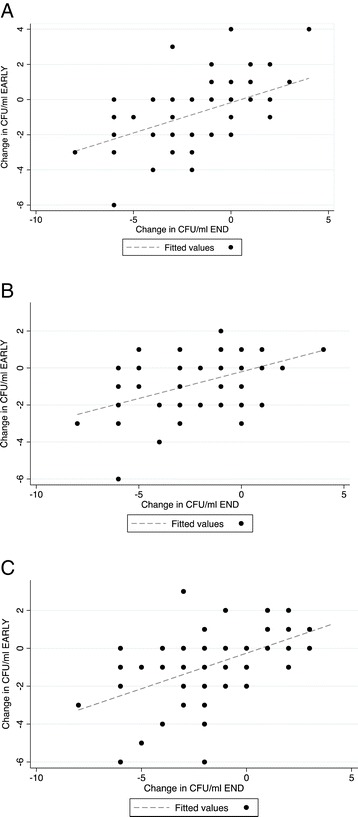
**Correlation in change in CFU from admission sputum sample Early in to exacerbation therapy (Y axis) to End of therapy (X axis). A)**. ALL *P. aeruginosa* morphotypes, **B)**. Mucoid only isolates, **C)**. Non-mucoid Isolates.

Change in CFU of *P. aeruginosa* EARLY into the antibacterial treatment correlated with change in load by the END of the treatment for ALL (Prob > F <0.0001, r^2^ = 0.27), MUC (Prob > F =0.0003, r^2^ = 0.189) and NON-MUC (Prob > F <0.0001, r^2^ = 0.30) (Figure 4). EARLY changes in bacterial load did not, however, predict failure risk for any *P. aeruginosa* morphotype (not shown).

## Discussion

PEx are increasingly recognized as a significant contributor to the progressive nature of CF lung disease [24]. Avoiding PEx is critical in this regard, and many CF therapies may exert their beneficial effects in manner [5,25-27]. However, despite attempts to limit their occurrence, PEx remain common. Given that some PEx are associated with worse outcomes than others, strategies to identify and optimize the management of these events are critical.

Anti-pseudomonal antibiotics are an integral component of PEx management. Independent of enhanced airway clearance, anti-pseudomonal antibacterials have been shown to improve pulmonary function in a linear capacity relative to reducing *P. aeruginosa* burden [18]. Few studies however, have tried to identify factors associated with enhanced *P. aeruginosa* killing. Smith et al., followed 75 patients chronically infected with *P. aeruginosa* through PEx managed with single or dual agent anti-pseudomonals [28]. While they observed a general reduction in sputum density of *P. aeruginosa*, reduced sputum DNA and protein levels, none of these factors correlated with absolute change in pulmonary function. Similarly, Mclaughlin et al., did not observe a correlation between burden of *P. aeruginosa* and absolute lung function recovery [29]. More recently, Deschaght et al., performed an observational study where they identified a correlation between improvement in lung function and reduction in *P. aeruginosa* density using quantitative PCR [30]. These studies, however, focused on aggregate analysis of lung function improvement and did not categorize individual events on the basis of success or failure, nor assess impact of individual morphotypes.

We sought to determine if bacteriologic killing as a measure of effectiveness of antibacterial therapies correlated with clinical outcomes in PEx. During antibacterial treatment of PEx, a progressive decline in *P. aeruginosa* burden was noted in approximately 60% of patients. This decline was predictable based on early changes *in P. aeruginosa* burden after only four days of treatment. Reduction in total *P. aeruginosa* burden, nor mucoid or non-mucoid isolates specifically did not, however, correlate with risk of failing to recover lung function. Enhanced killing of *P. aeruginosa* was observed in patients receiving antibacterials estimated to have more potent *in vitro* activity. This was particularly true when antibiotics had greater activity against non-mucoid isolates that were more readily reduced with antibiotics. Furthermore, we have observed like others, that in those few patients who transiently achieve clearance of *P. aeruginosa* during PEx treatments, no enhancement in clinical outcomes was afforded [29].

To complicate matters, studies have shown resolution of PEx in patients chronically infected with *P. aeruginosa* treated with regimens devoid of anti-pseudomonal activity suggesting response is more complicated than a one-host and one-pathogen model [31,32]. Accordingly, more recent studies have assessed the impact of antibacterials on other constituents of the CF respiratory microbiome, beyond classical CF pathogens. Using enhanced culturing to identify the *Streptococcus milleri* group (*S. anginosus* group, SMG), 40% of PEx were identified to have this organism emerge as numerically dominant at the onset of PEx, and resolution of symptoms correlated with reduction in sputum burden [31,33,34]. Using culture independent techniques, others have observed that with the exception of the *Prevotella* and *Chrysiogenales*, levels of other microbiome constituents do not correlate with improvements in lung function [35,36].

We did not detect an increase in either total *P. aeruginosa* load, or specifically MUC or NON morphotypes associated with an exacerbation. Nor was there an association between change in *P. aeruginosa* sputum density and extent of decline in pulmonary function. This data mirrors that of other recent studies that have challenged the long held belief that an increase in *P. aeruginosa* triggers PEx [37-39]. Clearly, factors other than changes in macroscopically apparent *P. aeruginosa* morphotypes are involved. These factors may include: environmental exposures such as pollution [40] and allergens [41]; host factors such as medication compliance [42] and co-morbidities [15,43,44] pathogen factors such as respiratory viruses [45], changes in the constituents of the lower respiratory microbiome [33,46] and specific *P. aeruginosa* sub-populations [47,48]. Furthermore, it is likely that there are multiple convergent pathways leading to a PEx, and these events vary in their manifestations and potential outcomes.

Many contentious issues exist in PEx management strategies. Data in these areas are difficult to interpret as outcomes have traditionally focused on absolute improvement in pulmonary function, as opposed to a more appropriate model of proportional recovery [46,47] Whereas some, but not all studies have suggested HPTP to be inferior, we noted similar outcomes in risk of failures and reductions in *P. aeruginosa* burden [46,47]. Likewise length of therapy for PEx has been debated [49]. Herein we noted a trend towards increased risk of failure to recover lung function with therapies ≤10 days, but no benefit with extending therapies >14 days. We also observed treatments <14 days were inferior in reducing *P. aeruginosa* sputum density.

The effectiveness of different antibiotics in achieving good PEx outcomes has been previously assessed. Herein we did observe differential abilities of antibiotics to reduce *P. aeruginosa* burden. In particular, cefepime was inferior at reducing *P. aeruginosa* sputum burden, although no impact on PEx outcome was noted. Interestingly, meta-analyses have demonstrated patients treated with cefepime relative to an alternate β-lactam with a similar spectrum of activity may be associated with an increased risk of mortality [50,51]. While commonly used in CF, the only clinical studies evaluating cefepime in CF have been pharmacokinetic based [52,53]. Based on these data, further studies of cefepime usage in CF are advisable.

ICS are common agents in CF, with approximately one third of patients receiving them. However, their utility has not clearly been established and the general consensus is that they are over used and may even be harmful [54]. Indeed, in a randomized controlled trial to evaluate the effects of discontinuation of ICS in patients stably maintained on ICS showed equivalent outcomes [55]. ICS use has been associated with increased risk of PEx [43,56]. Our results now suggest an increased risk of PEx failure with ICS use, a trend towards reduced sputum density of *P. aeruginosa* during antibacterial treatment and may warrant further caution with these agents in CF.

Herein we have attempted to expand upon other studies assessing PEx outcomes and changes in *P. aeruginosa* sputum density. To do so we have utilized a proportional lung function recovery model to overcome the deficiency whereby other studies using absolute improvement fail to account for the extent of initial decline and therefore are poor predictors of success or failure [15]. While this work relies on clinician diagnosed PEx events, we have retrospectively confirmed they met diagnostic criteria using an established PEx diagnostic algorithm [5]. Furthermore, we have attempted to evaluate outcomes based on individual morphotypes of *P. aeruginosa* reported routinely by clinical laboratories [20]. Indeed, quantitative analysis of *P. aeruginosa* using qPCR would not distinguish between mucoid and non-mucoid isolates. As infection with mucoid isolates of *P. aeruginosa* is associated with reduced success of eradication treatment in patients with new infection [57,58] and a worse prognosis for patients with chronic lung infection [3,59], it might be expected that a differential response against mucoid isolates might impact PEx outcomes. This appears not to be the case.

Several limitations of this work deserve consideration. Because diagnosis of PEx were collected and analyzed retrospectively, the data is not as robust as prospectively collected data. Symptoms or signs not documented in clinical records may inadvertently be documented as absent. This may be somewhat mitigated, as these findings were collected at a single CF clinic over a short time period, where practice patterns were relatively uniform. Our study excluded PEx not requiring parenteral antibiotics. We observed a significantly higher rate of PEx failures than in other works [10,11,15]. While similar criteria were used in the selection of only parenteral antibiotic treated PEx, far fewer patients were treated with parenteral antibiotics in this study. This likely reflects institutional practice variation in the management of PEx and availability of local resources. Parenteral antibiotics were reserved for the most severe events as evidenced by the significant decline in lung function on admission. This is further supported by the relatively low level of *P. aeruginosa* drug resistance observed herein relative to other works [15,60]. Whether more aggressive management of exacerbations would result in improved outcomes is of interest.

## Conclusions

In this work we identified that while the majority of patients treated with parenteral anti-pseudomonals experienced a temporary reduction in sputum burden, approximately 40% did not. This bacteriologic response could be predicted on the basis of sputum samples collected early into antibiotic treatment. Antibacterial treatments with a greater number of agents with *in vitro* predicted activity against all morphotypes, and in particular non-mucoid isolates was more likely to result in enhanced *P. aeruginosa* killing during PEx. However, patients who experienced enhanced killing of *P. aeruginosa* after completion of therapy were not afforded a reduced risk of PEx failure. Accordingly, bacteriologic response during PEx treatment cannot be used to predict success.

## Abbreviations

ALL: All *P. aeruginosa* morphotypes
CBA: Columbia blood agar
CF: Cystic fibrosis
CFU: Colony forming unit
CHOC: Chocolate agar
CI: Confidence intervals
CLSI: Clinical Laboratory Standards Institute
DTT: Dithiothreitol
EARLY: Sputum sample collected within 3 (+/−2) days of treatment initiation
END: Sputum sample collected at end of therapy (within 3 days of completion)
FEV_1_: Forced expiratory volume in one second
HPTP: Home parenteral therapy program
ICS: Inhaled corticosteroids
IQR: Inter-quartile range
MAC: MacConkey agar
MDR: multi-drug resistant
MSSA: Methicillin sensitive *Staphylococcus aureus*
MRSA: Methicillin resistant *Staphylococcus aureus*
ml: Milliliter
MUC: Mucoid morphotypes
NON: Non-mucoid morphotypes
OF: Oropharyngeal flora
OR: Odds ratio
PDR: Pan-drug resistant
PEx: Pulmonary exacerbations
S: Sensitive
SD: Standard deviation
TMP/SMX: Trimethoprim/sulfamethoxazole
TOB: Tobramycin
